# Impact of ASFV Detergent Inactivation on Biomarkers in Serum and Saliva Samples

**DOI:** 10.3390/pathogens11070750

**Published:** 2022-06-30

**Authors:** Lorena Franco-Martínez, Martin Beer, Silvia Martínez-Subiela, Edgar García-Manzanilla, Sandra Blome, Tessa Carrau

**Affiliations:** 1 Interdisciplinary Laboratory of Clinical Analysis, Interlab-UMU, Regional Campus of International Excellence ‘Campus Mare Nostrum’, University of Murcia, 30100 Murcia, Spain; lorena.franco2@um.es (L.F.-M.); silviams@um.es (S.M.-S.); 2 Moorepark Animal and Grassland Research Center, Teagasc, Irish Agriculture and Food Development Authority, P61 C996 Cork, Ireland; edgar.garciamanzanilla@teagasc.ie; 3 Institute of Diagnostic Virology, Friedrich-Loeffler-Institut, Suedufer 10, 17493 Greifswald-Insel Riems, Germany; martin.beer@fli.de (M.B.); tessa.carraugarreta@fli.de (T.C.)

**Keywords:** African swine fever, pathogenesis, biomarkers, serum, saliva, virus inactivation, detergent treatment, heat treatment, impact of treatment on biomarkers

## Abstract

African swine fever (ASF) is a notifiable viral disease of domestic and wild suids. Despite intensive research efforts, the pathogenesis of the disease is still far from being understood. Analysis of biomarkers in different body fluids may supplement traditional pathogenesis studies. As reliable protocols are often established in laboratories with lower biosafety, the reliable inactivation of samples is crucial. The objective of this study was to find a procedure that inactivates the virus while preserving the biomarkers for downstream analyses. To this means, three different inactivation protocols were employed, namely Tergitol-type NP-40 (NP-40), polyoxyethylene-p-t-octylphenol (Triton X-100) and one with 95 °C heating. It could be demonstrated that all samples treated with 0.5% (*v*/*v*) concentration of both detergents showed an absence of virus infectivity. The same was true for heated samples. However, heated serum was not suitable for analyses. Next, the impact of treatment on biomarker readouts was assessed. While all protocols had an impact on the detection of biomarkers, correlation was retained. In particular, NP-40 may be the desired detergent for more accurate measurements while achieving efficient virus inactivation. Based on these studies, samples can be reliably inactivated for most biomarker analyses, and thus broader interdisciplinary cooperation is possible.

## 1. Introduction

African swine fever virus (ASFV), an enveloped, double-stranded DNA virus belonging to the *Asfarviridae* family, is the causative agent of African swine fever (ASF), a highly lethal disease in domestic and Eurasian wild suids [1]. For many decades, ASF has been endemic in many sub-Saharan African countries and in Sardinia. Nonetheless, this scenario has radically changed after the introduction of the disease into Georgia in 2007, which was followed by the rapid spread of the virus to numerous eastern European countries, reaching the European Union in 2014 [1,2]. From that year onwards, recordings of infected wild boar and domestic pigs have been reported in the Baltic States, Poland, Germany, Romania, and Bulgaria, amongst others [3]. The virus has also spread to Asia and, more recently, to the Americas (OIE WAHIS visited online 13 February 2022). Consequently, the extent of ASF has now reached true pandemic dimensions [4].

Animals infected with ASFV show a panel of clinical signs that includes high fever, depression, and respiratory distress. In its worst disease course, ASF resembles a viral hemorrhagic fever with severe hemorrhages, lung edema, and neurological signs [5]. Pathomorphological changes include enlarged, hemorrhagic lymph nodes; the reddening of tonsils; congestion of the spleen or splenomegaly; petechiae in different organs such as the kidney, colon, or urinary bladder; and lung and gall bladder wall edema [2].

Unfortunately, the pathogenesis of the disease has not been fully elucidated and the gaps in knowledge hamper the development of safe and effective vaccines, which are still lacking [6]. To further our understanding of the disease, various biomarkers of the immune system, inflammation, muscle damage, stress, oxidative status, or anaerobic metabolism, among others, can be studied directly from the biofluids of affected animals [7]. Biochemical alterations have been described in ASF, such as an increase in serum acute-phase proteins [8,9]. Similarly, saliva has proven potential as a biofluid for ASF diagnostics [10] and as biomarkers of stress, inflammation, the immune system, or redox homeostasis, as described in Cerón et al. [11] and is actually being employed to evaluate porcine health and welfare non-invasively. However, these research activities are confronted with the problem that sample material from ASFV-infected animals can only be used under high containment conditions or must be subjected to reliable inactivation procedures prior to shipment.

Different virus inactivation protocols that are compatible with good performance in clinical chemistry and hematological analyses have been described for enveloped viruses [12], including inactivation through chemical non-ionic agents such as Tergitol-type NP-40 (NP-40) [13] or polyoxyethylene-p-t-octylphenol (Triton X-100) [10], which can also be combined with additional heat treatments [13].

In the search for a protocol that ensures reliable ASFV inactivation in biofluids while preserving the informative value of downstream biomarker analyses, i.e., biomarkers of inflammation, immune system parameters, stress, and redox homeostasis, the present study was conducted, using inactivation protocols with Triton X-100 or NP-40 and 95 °C heating.

## 2. Materials and Methods

### 2.1. Study Design

The study was conducted in three consecutive steps. Step one was carried out to show general inactivation of the ASFV with the chosen detergent or heating protocol. To ease initial validation, the cell-culture-adapted ASFV “ArmeniaΔ285LGFPhuCD4” (GFP-ASFV) variant was used. This virus isolate expresses green fluorescent protein (GFP) as a fluorescent marker [14,15]. By utilizing this virus, viral replication could be assessed through fluorescent readouts on a robust cell culture and sufficient high viral input for the different inactivation studies was guaranteed. Step two was conducted to transfer the inactivation protocols to the biological samples, i.e., serum and saliva. These samples were derived from animals experimentally infected with the wild-type ASFV “Armenia 2008”. Samples were taken at the humane endpoint at 7 days post inoculation. The third part aimed to assess the impact of the chosen protocols on relevant biomarkers. To this means, up to eleven biomarkers were measured in detergent-treated samples. A heat inactivation protocol using 95 °C for 10 min was added for comparison.

#### Animal Trial Samples

The sera and saliva samples originated from three domestic pigs that had been oro-nasally inoculated with 10^4^ 50% hemadsorbing units (HAD_50_) of the highly virulent genotype II ASFV strain “Armenia 2008”. The animal trial was approved by the competent authority, (Landesamt für Landwirtschaft, Lebensmittelsicherheit und Fischerei (LALLF), Rostock, Germany) under the reference number 7221.3-2-011/19. All samples were stored at −80 °C until use.

### 2.2. Detergent and Heat Treatments

In order to find the most suitable detergent concentration, a dilution-dependent inactivation experiment was performed. First, GFP-ASFV was subjected to different inactivation protocols. Subsequently, the methodology was transferred for use with the saliva and serum samples of ASFV-infected animals.

Each sample (GFP-ASFV, saliva, or serum) was divided into six aliquots and was subsequently treated. To this end, Triton X-100 (2% *v*/*v*; Sigma-Aldrich, St. Louis, MO, USA) and NP-40 (10% *v*/*v*; Thermo Fisher Scientific, Waltham, MA, USA) were further diluted to the following final concentrations: 0.5%, 0.1%, and 0.01% (*v*/*v*), and one aliquot of the chosen matrices was then exposed to one of the different detergent treatments. Residual virus infectivity was measured by a standard limiting dilution assay, expressed as HAD_50_/mL or Tissue Culture Infective Dose 50% (TCID_50_/mL). Three independent experiments, following the previously described workflow, were carried out in order to evaluate the exposure effect of both detergents on the infectivity of ASFV. Additionally, the heat inactivation experiment was also performed as a means of comparison. To this end, the sera and saliva samples were inactivated at 95 °C for 10 min in a water bath.

The best performing concentration was chosen, and the samples were divided into four aliquots and treated as follows: (*i*) no inactivation treatment; (*ii*) inactivation with a final concentration of 0.5% (*v*/*v*) NP-40, incubated for 60 min at room temperature (RT); (*iii*) inactivation with a final concentration of 0.5% (*v*/*v*) Triton X-100, incubated for 60 min at RT; (*iv*) heat inactivation at 95 °C for 10 min. Saliva and serum from the negative donors and cell culture medium were included as negative controls and were passaged in the same run. This inactivation workflow was carried out in both step one and step two.

### 2.3. Virus Isolation and Titration

The heat- and detergent-inactivated samples that were obtained after performing steps one and two were subsequently tested for virus isolation.

The samples obtained in step one were subjected to one blind passage in permanent WSL cells, whereas experiments using serum and saliva (step two) used primary porcine macrophages for this purpose. The GFP expression in WSL cells and hemadsorption on primary macrophages were used as a read-out system to quantify potential infectivity.

WSL cells were cultivated in Iscove’s Modified Dulbecco’s Medium with Ham’s F-12 Nutrient Mix (Thermo Fisher Scientific, Waltham, MA, USA): 10% FBS, 1% penicillin, and streptomycin (10,000 U/mL; Gibco, Thermo Fisher Scientific, Waltham, MA, USA). Virus isolation and titration on porcine macrophages were carried out with macrophages derived from peripheral blood mononuclear cells (PBMCs) as previously described [16].

For the blind passages, 5 × 10^6^ cells of either WSL cells or PBMCs per well were seeded into 24-well Primaria plates (Corning, Durham, NC, USA) one or two days before inoculation, respectively. Then, 100 μL of 5-fold serially diluted samples (the dilution factors ranged from 1:5 to 1:1600) were inoculated onto naïve WSL cells or macrophages. After 2 h incubation at 37 °C to allow virus adsorption, cells were washed once with PBS and fresh medium was added. Cultures were examined daily for cytotoxicity with a light microscope and were cultivated for 5 days at 37 °C. After a freeze-thaw cycle, virus titrations were carried out.

For proof of infectious virus, either 7.5 × 10^4^ PBMCs or 3 × 10^5^ WSL cells per well were seeded in a 96-well Primaria tissue culture plate (Corning, NY, USA). The following day, the attached cells were inoculated with 100 µL of supernatant from the blind or second passage in ten-fold dilutions from 10^−1^ to 10^−8^. One day after inoculation, 20 µL of a 1% solution of red blood cells was added to each well containing primary macrophages. Plates were examined after 72 h. Each well with at least one hemadsorbing macrophage or green fluorescence indicative of virus replication, as read under a light or epifluorescence microscope, respectively, was considered positive. Titers were calculated by the Spearman–Kärber method and were expressed as log_10_ HAD_50_ in serum, or as TCID_50_ in cell culture medium, with a detection limit of 10^1.75^ per mL.

### 2.4. Assessment of Cell Toxicity

Detergents can potentially produce high cytotoxicity in cell culture and can compromise the correct validation of the results. To this end, following the work of Tanneberger et al. [17], the respective concentrations of the used detergents (0.05%, 0.1%, and 0.5%) were first titrated without virus and added to the corresponding cell culture in 96-well plates. The cell culture was incubated and tested for toxicity over 2 h. If cell toxicity (necrosis/detachment of cells) was observed during this time in one or more dilution levels, the respective dilution level was added to a 24-well plate containing cell culture. Additionally, throughout the experiments, at least one control was titrated per experiment (a tube with the regular experimental setup and virus, but without the addition of detergent).

### 2.5. Effects of ASFV Inactivation in Different Biomarkers

After determination of the effective concentration to inactivate ASFV, the possible effects of ASFV inactivation protocols in a battery of relevant biomarkers were evaluated. For this, a total of ten paired porcine saliva and serum samples obtained from healthy animals were divided into four aliquots and treated as previously described in Section 2.2.

Cortisol and haptoglobin in the saliva samples were measured using an AlphaLISA assay validated for its use in porcine samples [7], and were expressed in ng/mL. All other analytes (amylase in serum (IU/L) and alpha-amylase in saliva (IU/mL)), adenosine deaminase (total: tADA and isoenzyme ADA2 (UI/L)), total protein (TP, g/dL in serum and mg/dl in saliva), cupric reducing antioxidant capacity (CUPRAC, mmol trolox equivalents/L), and ferric reducing ability of saliva (FRAS, which is the same as ferric reducing ability of plasma or FRAP, mmol/L), trolox equivalent antioxidant capacity using the horseradish peroxidase (TEACH, mmol trolox equivalents/L), gamma-glutamyl transferase (GGT, IU/L), lactate dehydrogenase (LDH, IU/L), and haptoglobin in serum (g/L) were measured in an automated biochemical analyzer (Olympus AU400, Olympus Diagnostica GmbH, Ennis, Ireland) using commercial assays which were previously validated for their use in porcine samples [18,19].

Normality in the distributions was evaluated using the D’Agostino and Pearson omnibus normality test, and data did not follow a normal distribution. To evaluate the effects of the different inactivation protocols on the biomarkers, data were assessed using a one-way ANOVA (Friedman multiple comparisons test). To determine correlations between the different methods in both serum and saliva, the Spearman’s correlation test was performed.

## 3. Results

### 3.1. Triton X-100 and NP-40 Produce Dilution-Dependent Inactivation

**Figure 1:**
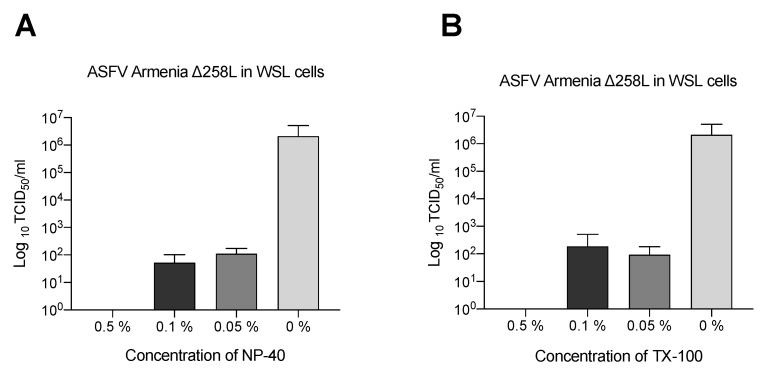
Inactivation of ASFV in cell culture supernatant by Triton X-100 and NP-40. ASFV containing cell culture supernatant was treated with three detergent dilutions ranging from 0.5% (*v*/*v*) to 0.0% (*v*/*v*) NP-40 (**A**) and Triton X-100 (**B**) for 60 min at RT. After the incubation period, both treated and untreated samples were back-titrated by limiting dilution on WSL cells. The mean value and standard deviation of duplicate tests are shown. Infectivity is expressed as Log TCID_50_/mL (detection limit of 10^1.75^ Log TCID_50_/mL).

To establish the optimal NP-40 and Triton X-100 concentrations, the WSL-adapted ASFV strain (ArmeniaΔ285LGFPhuCD4) was exposed for 60 min to dilutions ranging from 0.5% (*v*/*v*) to 0.01% (*v*/*v*) (Figure 1). A mean titer reduction of 5 Log TCID_50_/mL was observed for both detergents when compared to the untreated sample. The absence of virus infectivity was observed in 0.5% NP-40 (Figure 1A) and Triton X-100 (Figure 1B) treated samples (also showing a reduction in infectivity of 99.99%). After performing the dilutions as described above, no cytotoxic effects were observed. However, the cell culture was incubated and tested for toxicity over 2 h. If cell toxicity (necrosis/detachment of cells) was observed during this time in one or more dilution levels, the respective dilution level was added to a 24-well plate containing cell culture. Again, incubation and observations of toxicity were performed for 2 h.

**Figure 2:**
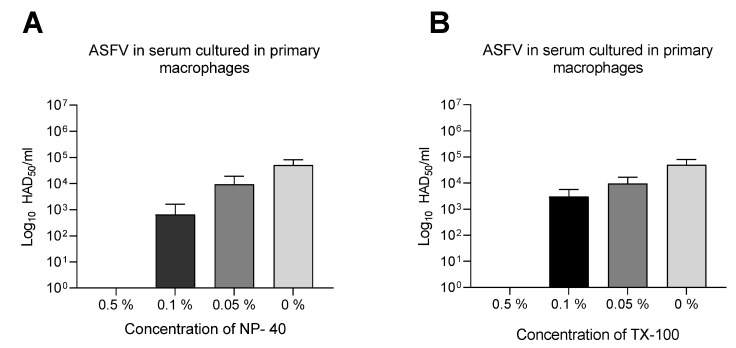
Inactivation of ASFV in serum by Triton X-100 and NP-40. ASFV containing sera were treated with three detergent dilutions ranging from 0.5% (*v*/*v*) to 0.1% (*v*/*v*) NP-40 (**A**) and Triton X-100 (**B**) for 60 min at RT. After the incubation period, both treated and untreated samples were back-titrated by limiting dilution on primary macrophages. The mean value and standard deviation of duplicate tests are shown. Infectivity is expressed as Log HAD_50_/mL (detection limit of 10^1.75^ Log HAD_50_/mL).

Subsequently, serum and saliva were used to test the impact of the matrix on the virus inactivation protocols. As shown in Figure 2A,B, 0.1% and 0.05% detergent concentrations revealed a similar efficacy in serum samples, with an average reduction of the virus titer of 2 Logs HAD_50_/mL compared to the untreated sample. This scenario differed when samples were treated with 0.5% (*v*/*v*) concentration (Figure 2A,B), where the absence of virus infectivity was notable in treated sera (≥6 log_10_ infectivity reduction). All saliva samples obtained for this study, including non-treated specimens, were negative for infectious virus.

### 3.2. Virus Infectivity

To determine whether 0.5% (*v*/*v*) Triton X-100 and NP-40 completely abolished infectivity after treatment, up to three passages were carried out for each of the treated samples. As summarized in Table 1, all detergent-treated samples were negative in the virus isolation assay and demonstrated no residual infectivity upon three consecutive passages of the inoculated cells. In contrast, the control samples that were not treated with detergents showed no obvious loss in virus titers. The effect of heat combined with the detergents was also tested alongside the detergents. Similarly, no infectivity was detected in any of these heat-treated samples upon three consecutive passages of the inoculated cells (Table 1), whereas the virus control retained the virus titer (Table 1). Saliva samples, including non-treated samples, showed a lack of infectivity during the experiment.

### 3.3. Effects of ASFV Inactivation on Different Biomarkers

The impact of ASFV inactivation protocols on each biomarker concentration is shown in Table 2. All sera heated at 95 °C were coagulated and were therefore not suitable for any measurements. In serum, TX-100 did not significantly affect ADA2, FRAP, nor LDH, whereas amylase, ADA, total protein, CUPRAC, TEACH, GGT, and haptoglobin were affected. The ASFV NP-40-based inactivation protocol did not affect cortisol, amylase, Hp, CUPRAC, TEACH, GGT, nor TP, but ADA, FRAP, and LDH showed differences compared to untreated aliquots.

In saliva, TX-100-based inactivation did not affect the levels of cortisol, alpha-amylase, Hp, ADA, CUPRAC, TEACH, and PT, whereas the levels of FRAS, GGT, and LDH were altered. Cortisol, alpha-amylase, Hp, ADA, CUPRAC, TEACH, GGT, and LDH were not affected by NP-40, opposing protein content and FRAS. Heating at 95 °C significantly changed the levels of all measured biomarkers, except for CUPRAC, TEACH, and TP. The correlations between untreated aliquots and TX-100, and NP-40 and 95 °C heating in saliva and serum are shown in Table 3. With the exception of amylase, tADA, ADA2, GGT, and LDH at 95 °C heating, all evaluated correlations were positive with coefficients >0.73 and were statistically relevant.

## 4. Discussion

African swine fever has become a pandemic threat to sustainable pig production. Furthermore, still, safe, and efficacious vaccines are lacking, and the knowledge of beneficial and detrimental host responses remains unclear. To join forces and to make use of interdisciplinary possibilities in sample analyses, virus inactivation procedures that do not have a critical impact on the parameters in question are needed.

Hence, our study set out to find inactivation protocols for biological samples intended for biomarker analyses. The effectiveness of heating, Triton X-100, and NP-40 for the inactivation of ASFV in porcine saliva and serum samples was evaluated. The samples were first subjected to different detergent concentrations and heating in order to evaluate which protocols successfully inactivate ASFV. Taken together, these results suggest that Triton X-100 or NP-40 at 0.5% (*v*/*v*) concentration are highly effective in eliminating the infectivity of both extracellular and intracellular ASFV particles. Then, these detergent concentrations and 95 °C heating protocols were tested in a battery of biomarkers that are applied in porcine medicine, including markers of stress, inflammation, the immune system, and oxidative status. The final inactivation protocols presented here agree with previously published data on the use of detergent treatment [13,20,21,22] to inactivate various viruses. These protocols have additional advantages in that they employ economical reagents and do not need expensive infrastructure.

Detergent treatments for potentially infectious matrices were introduced into the manufacturing processes of medical products more than 20 years ago [23] and have contributed to the overall safety of laboratory procedures. Additionally, when compared with heat inactivation procedures, detergents seem to have less significant impacts on the clinical chemistry and the analysis of hematology parameters [24]. Although NP-40 has been used less extensively for virus inactivation, Triton X-100 is a well-established method because of its lack of interference in the analysis of biofluids, such as human plasma and blood [25]. Hersberger et al., [26] additionally showed that the latter does not seem to interfere with the performance of most chemical and hematological assays; though, Rubio and Franco-Martínez [24] reported that its presence at 0.5% concentration might interfere with the colorimetric lamp. However, the possible influence of the detergents used in this study on the analysis of porcine serum biomarkers and/or analytical tests remains to be studied.

The evaluated biomarkers represent analytes used to evaluate the general health status of pigs. Biomarkers to evaluate stress (cortisol, salivary alpha-amylase), inflammation (haptoglobin), immune system parameters (adenosine deaminase), and oxidative status (TEAC, CUPRAC, FRAP/FRAS) were included. In addition, other biomarkers commonly employed—namely total protein, GGT, and LDH—were evaluated [11].

The addition of 0.5% (*v*/*v*) TX-100 to serum produced changes in eight of the evaluated biomarkers, namely amylase, tADA, protein content, CUPRAC, TEACH, GGT, cortisol, and haptoglobin. However, a strong positive correlation with NT (r > 0.95, *p* < 0.05) was found for amylase, tADA, GGT, and Hp, whereas cortisol, protein content, CUPRAC, and TEACH had correlation coefficients > 0.73 (*p* < 0.05). In saliva, TX-100 did not significantly alter the levels of analytes, with the exception of a reduction in FRAS and LDH, and an increase in GGT. In these cases, the correlation coefficients were r = 1, *p* < 0.001; r = 0.88, *p* < 0.01; and r = 0.92, *p* < 0.01, respectively. Therefore, although TX-100 caused changes in the majority of the biomarkers that were evaluated, especially in serum, the high correlation coefficients observed in relation to NT aliquots suggest that it is an adequate alternative to inactivate ASFV or other enveloped viruses in porcine samples.

NP-40 was the inactivation treatment that caused fewer changes in biomarker levels. In serum, it altered the levels of tADA, ADA2, FRAP, and LDH; whereas in saliva, total proteins and FRAS were the only biomarkers that changed significantly. However, the correlations in biomarkers that were observed between untreated and NP-40-inactivated samples were strong in all cases, with correlation coefficients r > 0.9 (with the exception of total protein in serum, which had a correlation coefficient of 0.85). Therefore, the addition of NP-40 to porcine and saliva samples would be the preferred method for the selected biomarkers.

Heating at 95 °C for 10 min is a rapid protocol that only requires a heating bath, and no additional reagents need to be added to samples. Heating at 95 °C caused all serum samples to coagulate, preventing their analyses. Therefore, this method is not recommended for serum samples, unless the solid state of the sample does not interfere with the techniques used for biomarker measurement. Heating at 95 °C causes a marked decrease in cortisol, haptoglobin, alpha-amylase, tADA, ADA2, TEACH, and LDH in saliva samples, reaching zero concentrations in most cases. For TEACH, heating causes an 18.3% median reduction, although data was strongly correlated with NT (r = 0.9636, *p* > 0.001). GGT concentration also decreased to 0 UI/L, although in this case, the difference was not considered significant. Although it was not statistically relevant, 95 °C heating produced a 20% median increase in total protein (r = 0.9636, *p* < 0.001), which may be associated with protein denaturalization caused by heat. Overall, 95 °C heating could be employed for the measurement of CUPRAC and FRAP with confidence. The strong correlation observed between NT aliquots and 95 °C heating for protein content and TEACH also allow their measurement and posterior correction. However, 95 °C heating is not recommended for the measurement of other biomarkers such as cortisol, haptoglobin, alpha-amylase tADA, and ADA2.

Based on our results, NP-40 is the inactivation protocol that causes fewer alterations in biomarker concentrations compared to untreated aliquots, and thus, could be evaluated for the first time for use with other biomarkers. However, Triton X-100 could also be a suitable inactivation treatment since its correlation coefficients with untreated aliquots are, in most cases, close to 1. Finally, inactivation with 95 °C for 10 min coagulates serum samples, preventing their measurement. In saliva, 95 °C heating for 10 min was the treatment that caused the most alterations in biomarker levels, although some biomarkers were not affected.

The use of serum and saliva samples from ASFV-infected domestic pigs allowed us to test the inactivation of ASFV in complex biological matrices. This suggests that these treatments could also be used in other biological matrices, such as blood and other biological fluids. During the present study we were able to report that, when used alone, Triton X-100 and NP-40 are able to rapidly inactivate the infectivity of a large-enveloped DNA virus, such as ASFV, at a concentration of 0.5% without interfering with the measurement of important porcine biomarkers. Because the methodology used in this study was highly effective, the methodology was considered sufficient to render these samples non-infectious. Hence, these protocols may mitigate the health risks for animals.

## 5. Conclusions

While we did observe the impacts of virus inactivation procedures on biomarker detection techniques, correlation still warrants analyses. The best combination from our study was NP-40 at a concentration of 0.5%; which produced significant changes in the levels of tADA, ADA2, FRAP, and LDH in serum, and total proteins and FRAS in saliva. Based on our data, future animal experiments should be supplemented with biomarker analyses.

## Figures and Tables

**Table 1:** Effects of detergent and heat treatments on ASFV infectivity.

Treatment	Infectious Titers before Treatment	Titers after Treatment	Titers after Virus Isolation at the 3rd Cell Passage
0.5% (*v*/*v*) Triton X-100			
ASFV in porcine serum (HAD_50_)	5.07 × 10^4^	negative	negative
ASFV in porcine saliva (HAD_50_)	negative	negative	negative
ASFV in culture medium (TCID_50_)	2.12 × 10^6^	negative	negative
0.5% (*v*/*v*) NP-40			
ASFV in porcine serum (HAD_50_)	5.07 × 10^4^	negative	negative
ASFV in porcine saliva (HAD_50_)	negative	negative	negative
ASFV in culture medium (TCID_50_)	2.12 × 10^6^	negative	negative

**Table 2:** Biomarkers after different ASFV inactivation treatments. For easier interpretation, results were normalized according to NT measurements, which were considered 100% and expressed as a median (25–75 percentile). Asterisk indicates differences of statistical relevance vs. NT measurements (* *p* < 0.05; ** *p* < 0.01; *** *p* < 0.001). NT: no inactivation treatment (control); NP-40: 0.5% NP-40; TX100: 0.5% Triton X-100; 95 °C: 95 °C heating for 10 min.

	Serum			Saliva			
	NT	TX-100	NP-40	NT	TX-100	NP-40	95 °C
Cortisol	100	112 (105–127) *	102 (98.7–112)	100	117 (88.7–155)	136 (104–235)	0 (0–0) *
Amylase/Alpha-amylase	100	91.6 (89.7–92.6) ***	99.2 (97.1–100)	100	90.8 (88.4–102)	95.3 (91.6–113)	0 (0–0) ***
Haptoglobin	100	83.5 (71.8–100) *	96.5 (88.7–113)	100	91.5 (81.7–119)	128 (114–182)	0 (0–0) **
tADA	100	91 (88.5–95.2) ***	93.7 (86.9–97.1) *	100	95.8 (91.5–98.5)	100 (93.9–104)	0 (0–0.1) ***
ADA2	100	108 (105–110)	112 (111–114) ***	100	97.4 (88.8–114)	103 (91.4–120)	0 (0–19.3) **
CUPRAC	100	118 (112–124) *	112 (110–118)	100	101 (95.5–113)	110 (103–112)	98.4 (94.7–101)
FRAP/FRAS	100	98.2 (96.9–99.8)	98 (93.8–98.8) **	100	90.1 (87.3–93.3) *	91.4 (88.5–94.2) *	98. (88.3–105)
TEACH	100	93.4 (90.3–95.8) ***	97.7 (94–100)	100	99.4 (94.9–101)	104 (94.9–108)	81.7 (78.3–90) **
GGT	100	97.8 (96–99.2) *	99.8 (97.9–101)	100	193 (131–288) *	123 (111–167)	0 (0–25) *
LDH	100	100 (97.8–103)	102 (102–104) *	100	56.2 (46.2–71.4) *	98 (88.3–101)	0.1 (0–2.4) ***
TP	100	93.5 (91.7–95.5) *	102 (100–103)	100	91.5 (86.7–94.5)	124 (118–134) *	120 (115–123)

**Table 3:** Correlation coefficients between different inactivation treatments and untreated samples for each biomarker. NT: no inactivation treatment (control); NP-40: 0.5% NP-40; TX100: 0.5% Triton X-100; 95 °C: 95 °C heating for 10 min. For easier interpretation, *p* < 0.05 are highlighted in bold.

	Serum		Saliva		
	TX-100	NP-40	TX-100	NP-40	95 °C
Cortisol	r = 0.879; *p* = 0.001	r = 0.867; *p* = 0.002	r = 0.4895; *p* = 0.11	r = 0.6294; *p* = 0.032	r = 0.0843; *p* = 0.895
Amylase/Alpha-amylase	r = 0.9879; *p* = 0.001	r = 0.9879; *p* = 0.001	r = 1; *p* = 0.001	r = 1; *p* = 0.001	r = 0.1741; *p* = 0.8
Haptoglobin	r = 0.9758; *p* = 0.001	r = 0.9879; *p* = 0.001	r = 0.9429; *p* = 0.017	r = 1; *p* = 0.003	r = 0.1243; *p* = 0.79
tADA	r = 0.9726; *p* = 0.001	r = 0.9605; *p* = 0.001	r = 0.9879; *p* = 0.001	r = 0.9515; *p* = 0.001	r = −0.2593; *p* = 0.4656
ADA2	r = 0.8831; *p* = 0.001	r = 0.9319; *p* = 0.001	r = 0.9205; *p* = 0.001	r = 0.9146; *p* = 0.001	r = −0.02055; *p* = 0.9558
CUPRAC	r = 0.7333; *p* = 0.02	r = 0.9879; *p* = 0.001	r = 0.9273; *p* = 0.001	r = 0.9515; *p* = 0.001	r = 0.9636; *p* = 0.001
FRAP	r = 0.8061; *p* = 0.007	r = 0.9515; *p* = 0.001	r = 1; *p* = 0.001	r = 0.9758; *p* = 0.001	r = 0.9785; *p* = 0.001
TEACH	r = 0.7939; *p* = 0.009	r = 0.903; *p* = 0.009	r = 0.9758; *p* = 0.001	r = 0.9636; *p* = 0.001	r = 0.9758; *p* = 0.001
GGT	r = 0.9273; *p* = 0.001	r = 0.9879; *p* = 0.001	r = 0.924; *p* = 0.001	r = 0.9024; *p* = 0.001	r = −0.1524; *p* = 0.716
LDH	r = 0.8061; *p* = 0.007	r = 0.9273; *p* = 0.001	r = 0.8788; *p* = 0.001	r = 0.9273; *p* = 0.001	r = 0.4282; *p* = 0.219
TP	r = 0.7815; *p* = 0.016	r = 0.8452; *p* = 0.006	r = 0.9636; *p* = 0.001	r = 0.9879; *p* = 0.001	r = 0.9879; *p* = 0.001

## Data Availability

Not applicable.
